# Description and molecular analysis of *Tylencholaimushelanensis* sp. n. from China (Dorylaimida, Tylencholaimidea)

**DOI:** 10.3897/zookeys.792.27255

**Published:** 2018-10-23

**Authors:** Wen-Jia Wu, Lu Yu, Hui Xie, Chun-Ling Xu, Jiao Yu, Dong-Wei Wang

**Affiliations:** 1 Lab of Plant Nematology/Research Center of Nematodes of Plant Quarantine, Department of Plant Pathology /Guangdong Province Key Laboratory of Microbial Signals and Disease Control, College of Agriculture, South China Agricultural University, Guangzhou, Guangdong 510642, China; 2 Key Laboratory of Vegetation Restoration and Management of Degraded Ecosystems, South China Botanical Garden, Chinese Academy of Sciences, Guangzhou, Guangdong 510160, China; 3 Grassland supervision and management of Alxa, Bayanhaote, Alxa Left Banner, Alxa League, Inner Mongolia 750399, China

**Keywords:** China, morphology, new species, phylogenetic analysis, taxonomy, *
Tylencholaimus
*

## Abstract

A new species, *Tylencholaimushelanensis***sp. n.**, extracted from the rhizosphere soil of unidentified grasses from Helan Mountain, Inner Mongolia, China was identified. The new species is characterized by having a body length of 0.93–1.07 mm with the lip region approximately one-quarter of the body diameter at the posterior end of the neck region wide; female didelphic-amphidelphic; pars proximalis vaginae violin-shaped. Males were not found. SEM observations of the new species were made and a phylogenetic analysis of both the 18S rDNA and the D2-D3 region of 28S rDNA is presented.

## Introduction

The genus *Tylencholaimus* de Man, 1876 is common in most soils all over the world and contains more than 50 valid species. It is mainly characterized by having small body, cap-shaped lip region, weak odontostyle and knobbed odontophore. The types of female genital system and tail of the genus are various (amphidelphic, monoprodelphic, or mono-opisthodelphic for female genital system, hemispherical to elongate-conical for tail) (Andrássy 2009). Peña-Santiago and Coomans (1994a, b, c, d; 1996a, b, c) revised the genus and its species, discussed the intrageneric variability and taxonomic value of some important morphological features such as the lip region, odontostyle, odontophore, pharynx, female genital system, tail and so on, and provided a key to the species. Peña-Santiago (2008) analyzed and discussed 15 species of *Tylencholaimus* described from 1996 to 2008, confirmed nine species to be valid, revised five species to be junior synonyms of other known species, transferred *T.annulatus* Baqri & Bohra, 2001 to the genus *Cricodorylaimus*, and provided an updated list and a key to the species of *Tylencholaimus*. Ahad and Ahmad (2016) added two new species to *Tylencholaimus*, redescribed six known species, and revised the diagnostic compendium and key to the species on the basis of Peña-Santiago (2008). In China, *Tylencholaimus* is widely distributed in many types of habitats such as mixed forest, broad-leaved forest, coniferous forest, alpine meadow, grassland, farmland, tea plantations, and wetland and others (Tong et al. 2009; Sang et al. 2010; Zhang et al. 2010; Wang et al. 2011; Xue et al. 2013; Hua et al. 2014; Xing et al. 2014; Yu et al. 2015). However, all the descriptions at the species level of these populations from China are lacking.

With detailed examinations based on light microscopy, SEM observations and phylogenetic analysis of 18S rDNA and the D2–D3 region of 28S rDNA, one nematode population from Inner Mongolia, China, was identified to be a new member of *Tylencholaimus* and is described as *Tylencholaimushelanensis* sp. n.

## Materials and methods

### Morphology and morphometrics

Soil samples were collected from the rhizosphere soil of unidentified grasses from Helan Mountain, Alxa Left Banner, Alxa League, Inner Mongolia, China. Nematode populations were extracted from the soil samples by using the modified Baermann funnel method (Whitehead and Hemming 1965). Then specimens were killed at 62 °C for 3 min, fixed in 4% FG fixative, dehydrated by using the glycerol-ethanol method, and then mounted on permanent slides (Xie 2005). The best preserved specimens were observed, photographed, and measured as described previously (Wu et al. 2016). For SEM observations, nematodes were prepared as described by Abolafia and Peña-Santiago (2005) and observed with a FEI XL-30-ESEM electron microscope at 10KV.

### DNA extraction, amplification, and sequencing

A single nematode was picked into 10 μL mixed solution (distilled water: 2×buffer for KOD FX = 1:1) and cut using a sterilized needle. 1 μL 20 μg/mL proteinase K was added and then reacted at 65 °C for 1 h and 95 °C for 15 min to release the genomic DNA. PCR reactions were performed in a 10 μL reaction mixture containing 5 μL of 2×buffer for KOD FX, 0.3 μL of each primer (10 μM) , 2 μL of dNTPs (200 μM), 1 μL of DNA, 1.2 μL of distilled water and 0.2 μL of KOD FX polymerase (1 U/ μL). Two overlapping fragments of the 18S rDNA were amplified using two primer sets, 988F (5'–CTCAAAGATTAAGCCATGC–3’) and 1912R (5'–TTTACGGTCAGAACTAGGG–3’) for the first fragment, and 1813F (5'–CTGCGTGAGAGGTGAAAT–3’) and 2646R (5'–GCTACCTTGTTACGACTTTT–3’) for the second one (Holterman et al. 2006; Nedelchev et al. 2014). For the D2-D3 region of the 28S rDNA amplifications, D2A (5’–ACAAGTACCGTGAGGGAAAGTTG–3’) and D3B (5’–TCGGAAGGAACCAGCTACTA–3’) (De Ley et al. 1999) were used. The PCR reactions were carried out as described previously (Wu et al. 2017). Electrophoresis was performed on 1% TAE agarose gels and observed under UV transillumination (AlphaImager® EP). Sequencing of PCR products was carried out by Sangon Biotech (Shanghai) Co. Ltd. The newly obtained sequences of the new species were deposited in GenBank (http://www.ncbi.nlm.nih.gov/Genbank/).

### Phylogenetic analysis

The sequences of the new species were compared with sequences in GenBank using BLAST. The sequences of other dorylaimid species and the outgroup taxa were chosen according to previous studies (Holterman et al. 2008; Nedelchev et al. 2014; Álvarez-Ortega and Peña-Santiago 2016), the sequences of other *Tylencholaimus* spp. were also included. The sequence alignment was done by using the software MEGA v.6 and the conservative regions were selected by using the online Gblocks server (http://molevol.cmima.csic.es/castresana/GBLOCKS_server.html). Substitution saturation was tested by DAMBE and the model of base substitution was evaluated using MrModeltest v2.3. The best-fit models were selected by AIC (Akaike Information Criterion) in MrModeltest v2.3. Phylogenetic trees were constructed by using MrBayes v3.1.2 running the chain for 5,000,000 generations for the 18S rDNA and 1,000,000 generations for the D2-D3 region of the 28S rDNA, respectively, with a sample frequency of 100 generations and setting the ‘burnin’ at 2500. The topologies were used to generate a 50% majority rule consensus tree with posterior probabilities (PP) for appropriate clades. The software Figtree v.1.3.1 was used to visualize and edit the phylogenetic trees.

## Results

### Tylencholaimus helanensis sp. n.

Taxon classificationAnimaliaDorylaimidaTylencholaimidea

http://zoobank.org/F0BFD129-C8C7-442C-8A1F-A7C144310D0D

Figs 1 , 2

#### Material examined.

Seven females from Qinghai Province; 38°40.311’N, 105°50.905’E; 22 August 2014; collected by Dong-Wei Wang, Wen-Jia Wu, Lu Yu, and Hui Xie. Female holotype (M51.B.a) and six female paratype specimens (slide numbers: M51.A.a, b, c, d, e and M51.B.b) are deposited in the Lab of Plant Nematology/Research Center of Nematodes of Plant Quarantine, South China Agricultural University, Guangzhou, Guangdong 510642, China.

**Figure 1.**
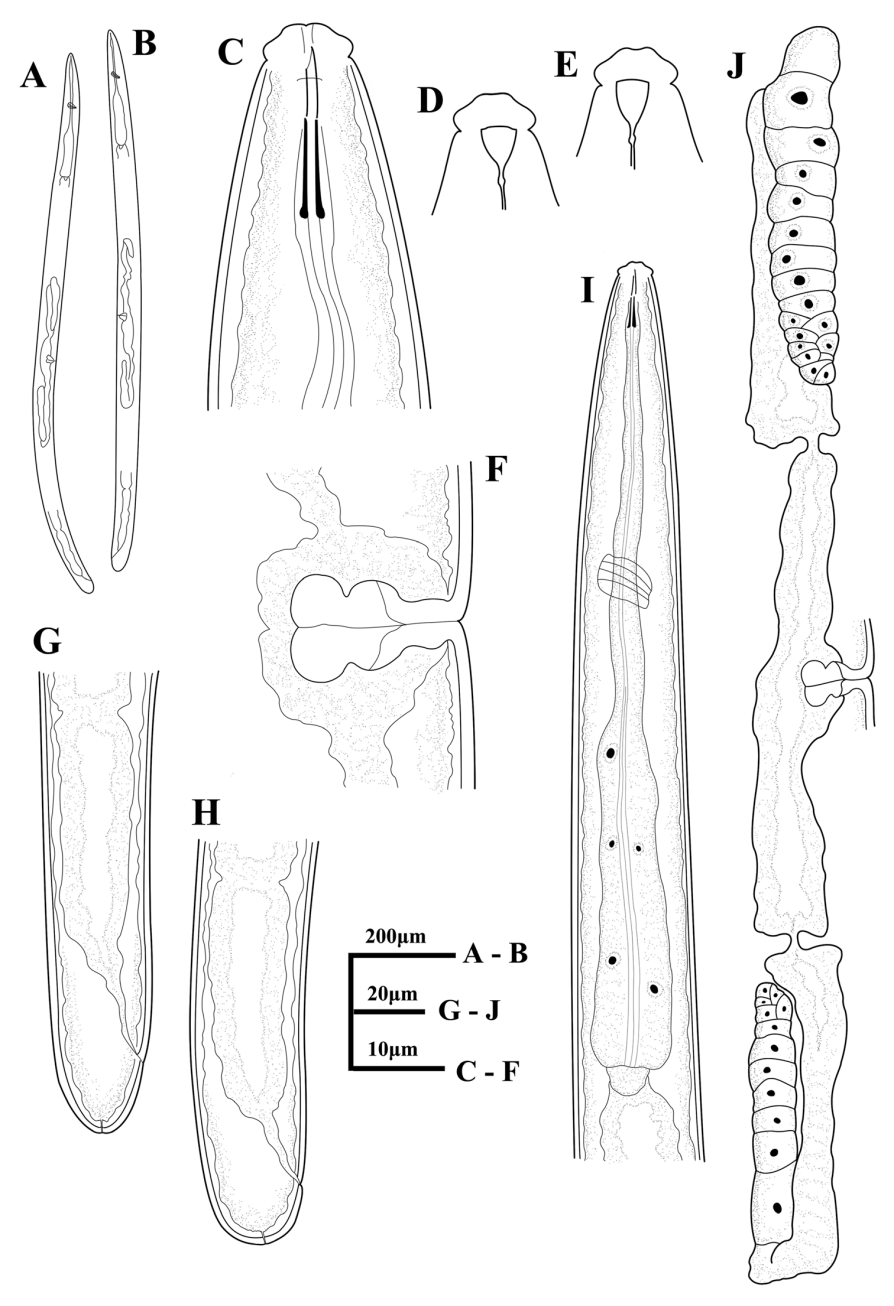
*Tylencholaimushelanensis* sp. n. Female: **A, B** entire body **C** anterior region showing odontostyle and odontophore **D, E** amphidial fovea **F** vulva in lateral view **G, H** posterior region **I** anterior region showing pharynx **J** genital system.

**Figure 2.**
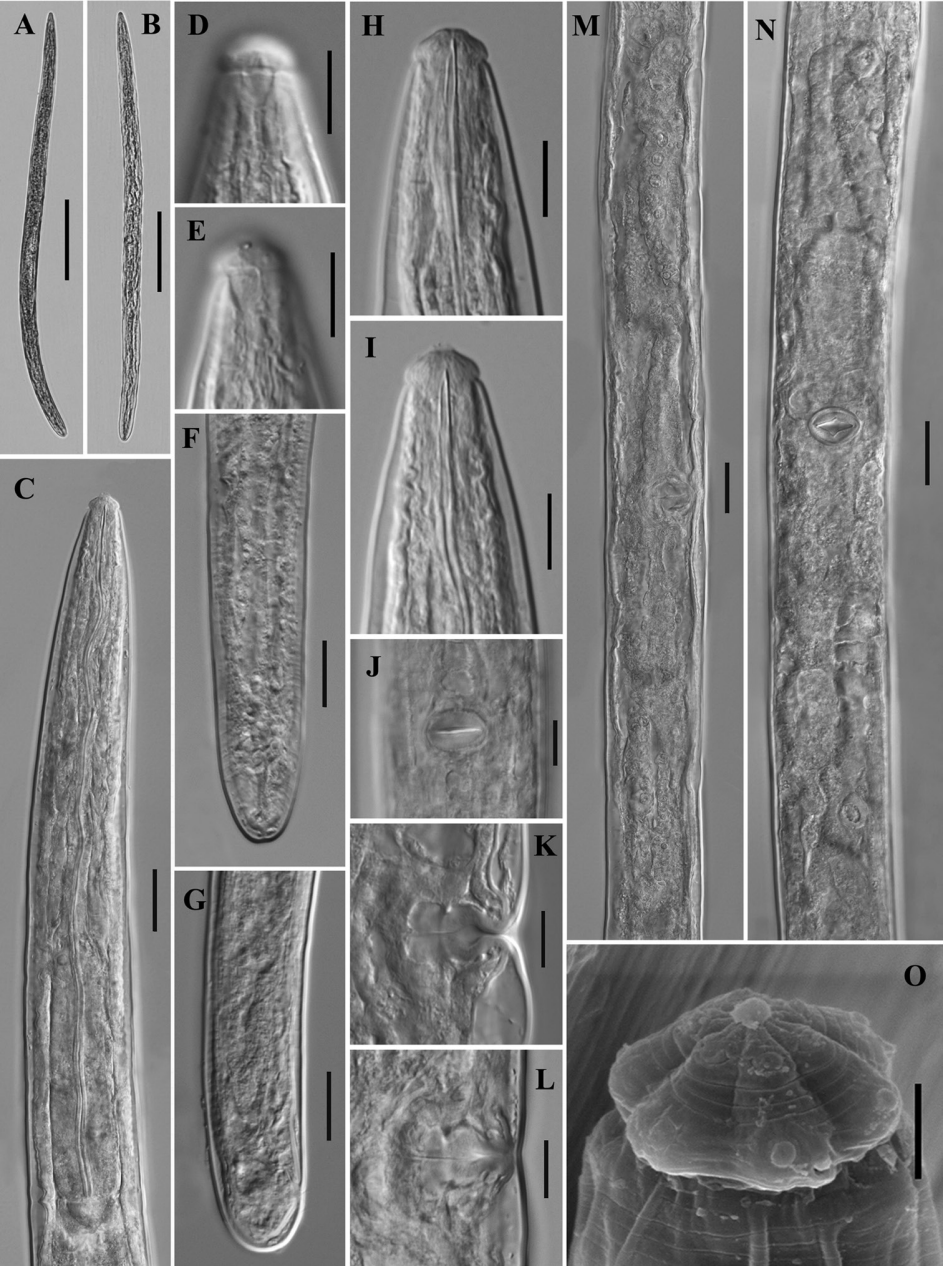
*Tylencholaimushelanensis* sp. n. Female: **A, B** entire body **C** anterior region showing pharynx **D, E** amphidial fovea **F, G** posterior region **H, I** anterior region showing odontostyle and odontophore **J**vulva in ventral view **K, L** vulva in lateral view **M, N** genital system **O** SEM of the lip region. Scale bars: 200 μm (**A, B)**; 20 μm (**C, F, G, M, N)**; 10 μm (**D, E, H–L**); 2 μm (**O)**.

#### Descriptions.

Female. Body robust and cylindrical, tapering towards the anterior end. Habitus variable, almost straight or slightly twisted after fixation. Cuticle two layers, 1.0–2.0 μm thick in anterior region, 1.5–2.5 μm at mid-body, and 2.5–3.5 μm on tail; outer layer with fine transverse striations, the inner one loose and often shrunken after fixation. Lateral chord occupying about one-third of the body diameter at mid-body, lateral pores indistinct. Lip region cap-shaped, offset from the body by a constriction, 2.4–2.8 times as wide as high or 25% in average of the body diameter at posterior end of the neck region wide. Lips not amalgamated, the outer part of each lip not distinct from the inner one. Labial and cephalic papillae distinct but not interfering with the contour. Amphidial foveae cup-shaped, opening at the level of the constriction, apertures 0.4 times on average as wide as the lip region. Odontostyle straight with a distinct lumen, 8–9.5 μm long, 0.9–1.0 times as long as the lip region width, its aperture about one-third of its length. Odontophore rod-like with small basal knobs, 9–11 μm long, 1–1.3 times as long as the odontostyle. Guiding ring single. Nerve ring situated at 35–42% of the neck length. Anterior part of pharynx slender and expanded gradually, basal expansion occupying 39–43% of the total neck length. Pharyngeal gland nuclei locations (Andrássy 1998) are as follows: D = 60–66%, AS1 = 21–30%, AS2 = 36–44%, PS1 = 62–74%, PS2 = 67–79%. Cardia short, conoid to rounded. Genital system didelphic-amphidelphic. Ovary reflexed, the anterior one 67–86 μm and the posterior one 54–79 μm long. Each oviduct consists of a wider *pars dilatata* and a slender part, 0.9–1.3 times the uterus long; anterior oviduct 83–107 μm and the posterior one 61.5–92 μm long. Sphincter present at the junction of oviduct and uterus. Uterus simple and with a wide lumen, the anterior one 66–85 μm and the posterior one 58–72 μm long. Vulva transverse. Vagina showing ‘+’ shape in ventral view, extending 44.5–46% inwards the corresponding body width. The walls of *pars* proximalis vaginae recessed inward in the middle, making pars proximalis vaginae violin-shaped, 12–13 μm long and 13–15 μm wide, with poorly developed musculature surrounding only the part adjacent to pars distalis vaginae. Pars refringens lacking, pars distalis vaginae 7 μm long. No sperm observed in the genital system. Prerectum 2.4–4.2 times and rectum 0.9–1.2 times the body diameter at anus level. Tail hemispheroid with blunt rounded to flat terminus. One caudal papilla opening in tail terminus.

Fore measurements see Table 1. The male was not found.

**Table 1.** Morphometrics of *Tylencholaimushelanensis* sp. n. and the females of its six close species. Measurements for *Tylencholaimushelanensis* sp. n. are in the form: mean ± s.d. (range), for other six species are in the form of range, and all in μm (except for ‘L’ in mm).

Character	*Tylencholaimushelanensis* sp. n.		* T. teres *	* T. congestus *	* T. cosmos *	* T. crassus *	* T. paracrassus *	* T. sinensis *
	Holotype	Paratypes	(1–4)*	(4, 5)*	(6, 7)*	(4, 5)*	(4)*	(8)*
n	1♀	6♀♀	>20♀♀	5♀♀	15♀♀	28♀♀	5♀♀	2♀♀
L	1.00	1.00 ± 0.06 (0.93–1.07)	0.8–1.06	0.72–0.83	0.57–0.9	0.68–0.92	0.9–1.1	0.76–0.82
a	26.3	25.6 ± 1.0 (24.8–27.5)	20–35.8	29.2–33	25.7–35	20–24	24–31	27–28.5
b	4.4	4.4 ± 0.4 (3.7–4.9)	4.0–5.1	3.2–4.2	3.7–5	3.2–4.7	4.0–4.8	3.6–4.3
c	54.5	51.3 ± 5.8 (46.2–60.7)	55.0–67.3	40–46	34.8–39.1	28–39	35–37	45–45.8
c’	0.6	0.7 ± 0.1 (0.6–0.8)	0.7–1.0	1	1.0–1.3	1	1.1–1.9	0.8–0.85
V	55.1	55.3 ± 1.2 (53.3–56.5)	51–66	60–62	57–63.4	52–57	52–58.5	57–57.5
G1	18.2	14.0 ± 3.2 (8.6–17.3)	18.4–27.9	17.3	9–24	–	–	18.5–19.5
G2	14.2	14.3 ± 1.8 (11.7–16.3)	13.9–21.5	17.0	10–17	–	–	16.5–18.5
Lip region diameter	10	10 ± 0.2 (9.5–10)	8–9.5	8	7–8	10.5–12	11.5–13	8
Lip region height	4	4 ± 0.2 (3.5–4.0)	4	3.5	2–3	5–5.5	–	3
Amphid aperture	4	4 ± 0.2 (3.5–4)	3–4	4	2	–	–	4–5
Odontostyle length	9	9 ± 0.4 (8–9.5)	5–6	7–8	7–8	8.5–9.0	10–11.5	7
Odontophore length	10	10 ± 0.7 (9–11)	8–9	8–9	9–14	9–11	–	8
Guiding ring from anterior end	6	5.8 ± 0.4 (5.5–6.5)	5–6	–	4–5.5	–	–	4–5
Nerve ring from anterior end	94	86 ± 6.6 (78–93)	72–90	80	63–71	–	–	73–83
Pharyngeal length	230	222 ± 8.5 (216–237)	202–244	193–220	146–207	211–222	213–249	191–208
Expanded part of pharynx	98	91.0 ± 6.4 (87–102)	81–110	76	61–87	90	90–106	67–75
Cardia length	12	11 ± 1.2 (9–12)	6–12	6	5–7	–	–	5–8
Body diameter at neck base	34	36 ± 4.1 (31.5–41.5)	24–31.5	26	19–24	–	–	–
Body diameter at mid-body	38	39 ± 2.7 (36–43)	26–34.5	28	19–26	–	–	–
Body diameter at anus	28	25 ± 1.3 (24–27)	19–22	18	15–21	28	21–25	–
Anterior genital branch	182	142 ± 38.2 (80–185)	172–265	141	58–95	–	–	142–164
Posterior genital branch	142	144 ± 16.3 (117–161)	134–219	139	68–87	–	–	125–155
Vaginal depth	14.5	19.0 ± 1.0 (18–20)	11–16.5	13	8–11	–	–	–
Vulva from anterior end	552	559 ± 34.8 (513–607)	553–658	492	345–420	–	–	440–474
Prerectum length	68.5	85 ± 12.9 (71–100)	69–200	94	22–70	–	47–66	100–105
Rectum length	26	25 ± 2.7 (22–28)	17–23	18	13–25	–	–	18–20
Tail length	18	19 ± 1.5 (16.5–21)	14–19	18	16–22	28	30–38	17–18

* References: (1) Loof 1971; (2) Thorne 1974; (3) Vinciguerra 1986; (4) Peña-Santiago and Coomans 1994a; (5) Loof and Jairajpuri 1968; (6) Dhanam and Jairajpuri 1999; (7) Ahad and Ahmad 2016; (8) Li et al. 2008.

#### Sequence and phylogenetic analysis.

**Figure 3.**
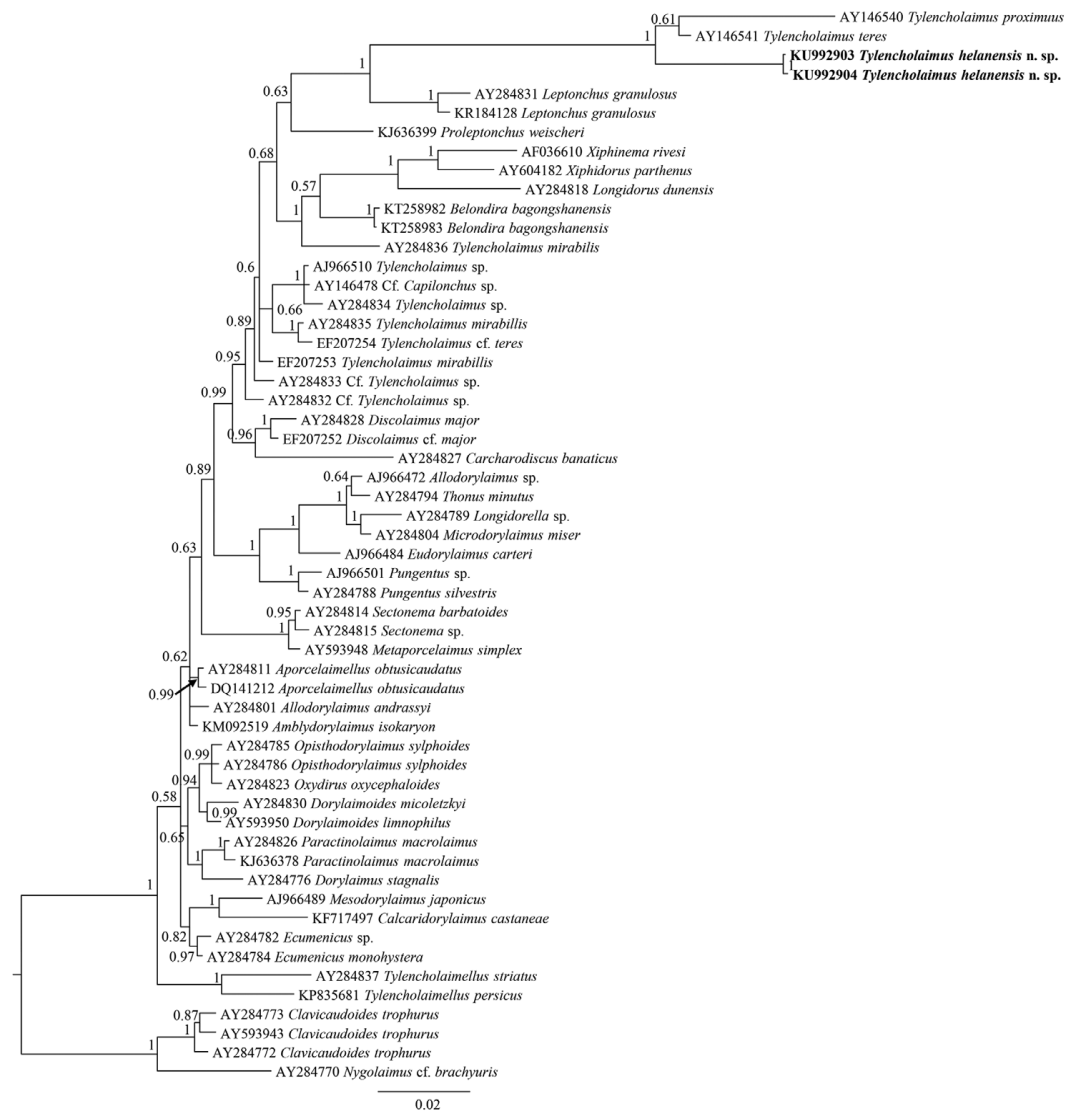
Phylogenetic relationships of *Tylencholaimushelanensis* sp. n. and other Dorylaimida species for 18S rDNA. Bayesian inference strict consensus tree is acquired under GTR+I+G model. Posterior probabilities higher than 50% are presented. Newly obtained sequences are given in bold.

**Figure 4.**
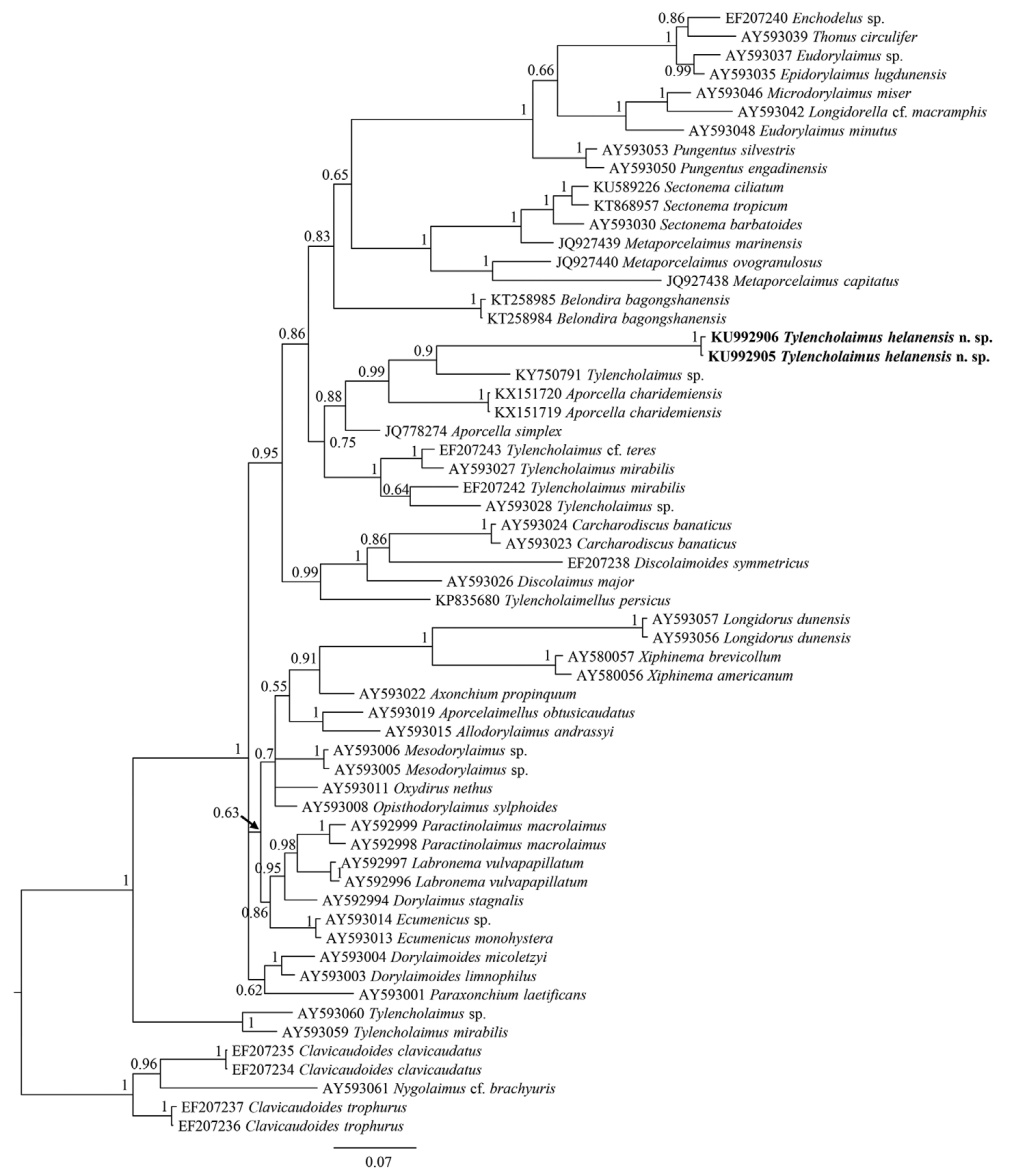
Phylogenetic relationships of *Tylencholaimushelanensis* sp. n. and other Dorylaimida species for the D2-D3 region of 28S rDNA. Bayesian inference strict consensus tree is acquired under GTR+I+G model. Posterior probabilities higher than 50% are presented. Newly obtained sequences presented in bold.

The sequences of 18S rDNA and D2-D3 region of 28S rDNA of *Tylencholaimushelanensis* sp. n. were obtained. The inter-individual variabilities of the 18S rDNA sequences and the 28S rDNA sequences are one gap and two base pair differences, respectively. Two sequences for each of the genes were deposited in GenBank (accession numbers: KU992903 (1746 bp long) and KU992904 (1747 bp long) for 18S rDNA, KU992905 and KU992906 (both 840 bp long) for D2-D3 region of 28S rDNA). The BLAST search for the 18S rDNA showed the highest similarity (94% and 95%) to the sequence of an unidentified species of *Tylencholaimus* (AJ966510). For the D2-D3 region of 28S rDNA, both sequences showed the highest similarity (79%) to the sequences of *Xiphinemabrevicollum* Lordello & Da Costa, 1961 (AY580057). In the 18S rDNA phylogenetic reconstructions (Fig. 3), the new species is in a 100% supported clade with *T.teres* and *T.proximus*. And in the D2-D3 region of 28S rDNA phylogenetic reconstructions (Fig. 4), the new species is in a clade with an unidentified species of *Tylencholaimus* with 90% posterior probability.

#### Type habitat.

Rhizosphere soil of unidentified grasses from Helan Mountain, Alxa Left Banner, Alxa League, Inner Mongolia, China.

#### Etymology.

The new species is named after the mountain Helan, which is a famous mountain with a wealth of human history including rock paintings, architecture, vineyards, and a national park.

#### Diagnosis and relationships.

*Tylencholaimushelanensis* sp. n. is characterized by having a body length of 0.93–1.07 mm; body tapering towards the anterior end; lip region offset from the body by a constriction and 25% in average of the body diameter at posterior end of the neck region wide; amphid aperture 0.4 times in average as wide as the lip region; odontostyle 8–9.5 µm long and 0.85–1.0 times as long as the lip region width; odontophore 1–1.3 times as long as the odontostyle; basal expansion of pharynx 39–43% of the total neck length; female genital system didelphic-amphidelphic; vulva transverse; prerectum 2.4–4.2 times and rectum 0.9–1.2 times the body diameter at anus long; tail hemispheroid with blunt rounded to flat terminus; males not found.

*Tylencholaimushelanensis* sp. n. is close to *T.congestus* Loof & Jairajpuri, 1968, *T.cosmos* (Dhanam & Jairajpuri, 1999) Peña-Santiago, 2008, *T.crassus* Loof & Jairajpuri, 1968, *T.paracrassus* Monteiro, 1970, *T.sinensis* Li, Baniyamuddin, Ahmad & Wu, 2008 and *T.teres* Thorne, 1939 in having a body length about 1 mm or less, female genital system didelphic-amphidelphic, odontostyle less than 10 μm and ‘V’ value less than 62 in average, but can be differentiated by having panduriform *pars proximalis vaginae*. In addition, the new species differs from *T.congestus* (Loof and Jairajpuri 1968; Peña-Santiago and Coomans 1994a) by having longer body (0.93–1.07 mm vs. 0.72–0.83 mm), lower ‘a’ value (a = 24.8–27.5 vs. 29–33), different lip region (lip region cap-shaped, lips not amalgamated and no inner liplets vs. lips apparently separated, inner part protruding and forming liplets), absence of large cells in the vaginal area (vs. presence) and oviducts 0.9–1.3 (vs. 3–4) times the uterus long. From *T.cosmos* (Dhanam and Jairajpuri 1999; Ahad and Ahmad 2016), the new species differs by having longer pharynx and basal expansion (216–237 μm vs. 146–207 μm; 87–102 μm vs. 61–87 μm, respectively), and sphincter present at the junction of oviduct and uterus (vs. uterus and oviduct without distinct sphincter differentiation). From *T.crassus* (Loof and Jairajpuri 1968; Peña-Santiago and Coomans 1994a) by longer body (0.93–1.07 mm vs. 0.68–0.92 mm), smaller lip region (9.5–10 μm vs. 10.5–12 μm wide; 3.5–4.0 μm vs. 5–5.5 μm high), absence of postrectal blind sac (vs. presence) and tail hemispheroid with blunt rounded to flat terminus (vs. convex conoid with rounded tip). From *T.paracrassus* (Peña-Santiago and Coomans 1994a), the new species can be differentiated by having narrower lip region (9.5–10 μm vs. 11.5–13 μm wide), shorter odontostyle (8–9.5 μm vs. 10–11.5 μm), longer prerectum (71–100 μm vs. 47–66 μm), tail hemispheroid with blunt rounded to flat terminus (vs. convex conoid with rounded tip) and males absent (vs. present). It differs from *T.sinensis* (Li et al. 2008) by lip region one-fourth (vs. one-third) of the body diameter at posterior end of neck region, longer odontostyle and odontophore (8–9.5 μm vs. 7 μm; 9–11 μm vs. 8 μm, respectively), longer pharynx and basal expansion (216–237 μm vs. 191–208 μm; 87–102 μm vs. 67–75 μm and ocuupying 39–43% vs. 35–36% of the total neck length, respectively), much longer oviducts (anterior one 83–107 μm vs. 53–63 μm and the posterior one 61.5–92 μm vs. 45–50 μm long), prerectum 2.4–4.2 (vs. about 5) times the body diameter at anus long, longer rectum (22–28 μm vs. 18–20 μm). From *T.teres* (Loof 1971; Thorne 1974; Vinciguerra 1986; Peña-Santiago and Coomans 1994a), it differs by the females having lip region one-fourth in average (vs. one-third) of the body diameter at posterior end of the neck region, odontostyle longer (8–9.5 μm vs. 5–6 μm), one caudal opening in tail terminus (vs. one pair of subterminal pores), the anterior and posterior genital branch equally developed (vs. the anterior branch more developed than the posterior one), no sperm observed in the genital tract and males not known (vs. sperm present along the entire genital tract and males as frequent as females).

## Discussion

In addition to the above characteristics used to differentiate the new species from its conspecifics, the pars proximalis vaginae of the new species should be noticed. Among the known didelphic species of *Tylencholaimus*, a cylindrical, spindle, convex, or pyriform pars proximalis vaginae has been described or illustrated. The violin-shaped structure in *Tylencholaimushelanensis* sp. n. is described here for the first time. This enriches the diversity of the pars proximalis vaginae and makes this characteristic more valuable for identification. In fact it is so distinctive that in the 18S rDNA and 28S rDNA Bayesian trees, *Tylencholaimushelanensis* sp. n. forms a monophyletic clade with 100% support. In the 18S rDNA tree, *Tylencholaimushelanensis* sp. n. is sister to a clade including *T.teres* and *T.proximus*. As mentioned previously, *Tylencholaimushelanensis* sp. n. is close to *T.teres* in morphology, but differs from the latter by several morphological characteristics such as a wider amphid aperture, a shorter prerectum, longer odontostyle and tail, and the fragments of their 18S rDNA sequences in common showed ten nucleotide differences. The new species does not otherwise show close relationships to *T.teres* in the 28S rDNA Bayesian trees, while the other close relative inferred from the 18S rDNA Bayesian tree, *T.proximus*, has a prodelphic genital system that is different to the didelphic-amphidelphic genital system of *Tylencholaimushelanensis* sp. n., and thus can be easily differentiated from the new species morphologically.

The sequences of *Tylencholaimus* species were not all grouped together in one clade in both the 18S rDNA and 28S rDNA Bayesian trees, suggesting that *Tylencholaimus* is not monophyletic. The deeper evolutionary relationships among *Tylencholaimus* currently cannot be further clarified due to because the few molecular data available for *Tylencholaimus*, especially 28S rDNA sequences, available on GenBank. For example, the relationship of the new species and *T.proximus* inferred from the 18S rDNA Bayesian tree was close, but this relationship cannot be confirmed because the 28S rDNA sequence of *T.proximus* is unavailable. Thus, the detailed relationships of *Tylencholaimus* species cannot be further resolved until more molecular data of *Tylencholaimus* are obtained.

## Supplementary Material

XML Treatment for Tylencholaimus helanensis
